# Editorial: MicroRNAs in endocrinology and cell signaling

**DOI:** 10.3389/fendo.2022.1118426

**Published:** 2022-12-19

**Authors:** Chun Peng, Julang Li

**Affiliations:** ^1^ Department of Biology, York University, Toronto, ON, Canada; ^2^ Department of Animal Biosciences, University of Guelph, Guelph, ON, Canada

**Keywords:** microRNA, endocrinology, cell signaling, gene regulation, circular RNA

Since the discovery of the first microRNA (miRNA) in *C. elegans* (1), our understanding of miRNA biology has been constantly expanding. It is now well-established that miRNAs play key roles in regulating gene expression and thereby being critically involved in the proper functioning of cells, tissues, and organisms. The role of miRNAs in the endocrine system and cellular signaling events has also been clearly revealed. They regulate the development of endocrine glands, control hormone production and secretion, and modulate the activity of hormones by affecting their receptors and intracellular signaling networks. Conversely, hormones and various cellular signaling pathways also regulate miRNA biogenesis. Finally, miRNAs are detected in body fluids and are proposed to have hormone-like activities (2–7). The proper production and activity of miRNAs ensure the normal functioning of organisms while their dysregulation is associated with the development of diseases.

Many studies have reported that miRNAs regulate the differentiation, proliferation, and apoptosis of hormone-producing cells. For example, miRNAs play important roles in modulating pancreatic β cell differentiation, growth and survival, and dysregulation of miRNAs has been observed in diabetic patients (8). Similarly, miRNAs regulate thyroid follicular cell proliferation and differentiation, while aberrant expression of miRNAs contributes to the development of diseases, such as goiter and thyroid cancer (9).

MicroRNAs alter the production and secretion of hormones, growth factors, and other intercellular signaling molecules. For peptide hormones, miRNAs can directly target the genes encoding signaling molecules (10, 11) or indirectly by targeting genes that control their production (12). They can also target genes involved in exocytosis and therefore affecting the secretion of hormones, such as insulin (13). For non-peptide hormones, miRNAs regulate the expression of enzymes involved in hormone production or degradation. For example, several miRNAs have been reported to regulate aromatase expression and thereby affecting estradiol production (14, 15).

MicroRNAs are major regulators of intracellular signaling events. They regulate the levels and/or activation of receptors and downstream mediators of hormones, growth factors, and other signaling molecules. For example, many miRNAs are known to target androgen, estrogen, and progestin receptors (16). Cellular signaling pathways, such as TGFβ/SMAD (17, 18), MAPK (19), Wnt/β-catenin (20), PI3K/AKT (21) and others (22, 23) are extensively modulated by miRNAs. On the other hand, many hormones and intracellular signaling pathways can also regulate miRNA levels. For instance, several pituitary hormones have been shown to regulate miRNA production in the adrenal glands, gonads (24), and liver (25). Many well-conserved intracellular signaling pathways, such as AKT (26), MAPK (27), and TGFβ/SMAD (28) have all been reported to regulate miRNA production. Thus, the interplay among miRNA, hormones, and signaling pathways is critical in regulating cellular processes.

In this Research Topic, we collected 7 papers that show how miRNAs affect the endocrine system and cellular signaling events. Pan et al. demonstrated that miR-574 inhibits ERK1/2 activation by targeting tissue inhibitor of metalloproteinase 3 (TIMP3), resulting in increased estradiol production from pig granulosa cells. This work provides an example of how a miRNA modulates a cellular signaling pathway to regulate hormone production. Shan et al. showed that miR-218-5p, which promotes trophoblast differentiation and uterine spiral artery remodeling by targeting transforming growth factor β2 (TGFβ2) (10), also targets a downstream mediator of TGFβ, SMAD2, and this leads to the induction of interleukin 1β (IL1β). This study illustrates how a miRNA targets multiple components of a signaling cascade and mediates crosstalk between different pathways. The TGFβ signaling may also be regulated by miR-33a-5p, as shown by Li et al.. This study identified carnitine O-octanoyltransferase (CROT) as a gene that exerts anti-tumor and paclitaxel-sensitizing effects in ovarian cancer cells. Interestingly, CROT negatively regulates SMAD2 and SMAD4, and is directly inhibited by miR-33a-5p, suggesting the possibility that miR-33a-5p can enhance TGFβ signaling. The study by Cai et al. offers an example that miRNAs may serve as biomarkers for diseases of endocrine glands. In this study, the authors systematically analyzed the diagnostic value of miR-221 and miR-222 for papillary thyroid cancer and found that the two miRNAs have the potential to be used as diagnostic markers. Further analyses of the target genes of miR-221 and miR-222 revealed that they may potentially regulate many signaling pathways. Using RNA-seq, Werry et al. identified sperm miRNAs that are differentially expressed between high- and low-fertility bulls. Gene set enrichment analysis suggested that these miRNAs may impact a variety of cellular pathways. Deng et al. reported that a psychoactive drug, methamphetamine, upregulates miR-129-1-3p to induce dopaminergic cell apoptosis and many of the predicted targets are involved in various cellular pathways and processes. They further demonstrated that miR-129-1-3p is inhibited by a circular RNA, circ_001589. Finally, Yan et al. showed that circ_0011707 is significantly downregulated in subjects with impaired fasting glucose and is further decreased in patients with type II diabetes, while miR-144-3p exhibits an opposite pattern. The authors further revealed that circ_0011707 can sponge miR-144-3p. Since miR-144-3p is known to target the gene encoding the glucocorticoid receptor, *NR3C1* (29), circ_0011707 likely affects glucocorticoid signaling by limiting the availability of miR-144-3p within cells.

## Author contributions

All authors contributed to the article and approved the submitted version.
